# Street-Level Bureaucracy and Documentation: Addressing Conflicting Rights in Nursing Homes through Care Plans

**DOI:** 10.1093/geroni/igaf122.2662

**Published:** 2025-12-31

**Authors:** Angela Perone

**Affiliations:** University of California, Berkeley, Berkeley, California, United States

## Abstract

Conflicting rights are exceedingly common in nursing homes, one of the most heavily regulated industry in the United States (e.g., safety, autonomy, privacy). Staff have increasingly incorporated documentation in resident care. This study investigates how do staff use documentation to address conflicting rights in nursing homes? It employs a qualitative comparative case study with 80 in-depth interviews of staff and ethnographic observation of interactions among staff, residents, and visitors for eight months at two nursing homes. Staff referenced “care plans” as the primary tool to address conflicting rights. Front-line workers (e.g., CNAs, floor nurses) used care plans as deferential problem-solving tools and deferred to “looking in the care plan” to identify what they should do. Mid-level managers used care plans as dynamic clinical problem-solving tools for residents by identifying how to shape the care plan with various team members, including ways to change medical orders. Upper-level managers employed care plans as a liability problem-solving tool focused on the staff and organization by emphasizing the importance of documenting decisions in the care plan to insulate workers and the nursing home against liability. Care plans in this study reflect ways in which nursing home staff leveraged the documentation burdens that many often loathed to address an often vague and ambiguous policy space in nursing homes: how to navigate conflicting rights. Care plans, thus, transformed a dreaded documentation tool into a problem-solving tool that provided some staff a sense of agency and direction—albeit in different ways among different staff levels.

